# Impaired Spatial Memory and Enhanced Habit Memory in a Rat Model of Post-traumatic Stress Disorder

**DOI:** 10.3389/fphar.2017.00663

**Published:** 2017-09-22

**Authors:** Jarid Goodman, Christa K. McIntyre

**Affiliations:** School of Behavioral and Brain Sciences, University of Texas at Dallas, Richardson TX, United States

**Keywords:** single prolonged stress, anxiety, extinction, multiple memory systems, hippocampus and memory, dorsolateral striatum, PTSD

## Abstract

High levels of emotional arousal can impair spatial memory mediated by the hippocampus, and enhance stimulus-response (S-R) habit memory mediated by the dorsolateral striatum (DLS). The present study was conducted to determine whether these memory systems may be similarly affected in an animal model of post-traumatic stress disorder (PTSD). Sprague-Dawley rats were subjected to a “single-prolonged stress” (SPS) procedure and 1 week later received training in one of two distinct versions of the plus-maze: a hippocampus-dependent place learning task or a DLS-dependent response learning task. Results indicated that, relative to non-stressed control rats, SPS rats displayed slower acquisition in the place learning task and faster acquisition in the response learning task. In addition, extinction of place learning and response learning was impaired in rats exposed to SPS, relative to non-stressed controls. The influence of SPS on hippocampal spatial memory and DLS habit memory observed in the present study may be relevant to understanding some common features of PTSD, including hippocampal memory deficits, habit-like avoidance responses to trauma-related stimuli, and greater likelihood of developing drug addiction and alcoholism.

## Introduction

Emotional arousal has a dramatic impact on the function of memory systems in the mammalian brain. The memory systems that have been examined in this regard include a cognitive spatial memory system dependent on hippocampal function and a stimulus-response (S-R)/habit system dependent on function of the dorsolateral striatum (DLS). In studies employing animals (e.g., rats and mice) and human subjects, very high levels of emotional arousal have been associated with impairments in hippocampus-dependent spatial memory and enhancements in DLS-dependent habit memory (for reviews, see Packard and Goodman, 2012, 2013; Schwabe, 2013; Goodman et al., 2017a). In addition, in learning situations that can be solved with either spatial or habit memory, stress biases animals and humans toward the use of a DLS-dependent habit strategy (Packard and Wingard, 2004; Schwabe et al., 2007, 2008; Elliott and Packard, 2008; Dias-Ferreira et al., 2009).

Several investigators have suggested that findings pertaining to the emotional modulation of memory are relevant to understanding a variety of human psychopathologies, in particular those involving high levels of stress and anxiety (e.g., McGaugh, 2004; Schwabe et al., 2011; Goodman et al., 2014; Goodman and Packard, 2016a,b). For instance, subjects with post-traumatic stress disorder (PTSD) demonstrate impairments in spatial memory function (Tempesta et al., 2012; Smith et al., 2015; Miller et al., 2017), as well as heightened avoidance responses to trauma-related stimuli (e.g., running away from a loud noise), which may be viewed as an exemplar of enhanced stimulus-response (S-R)/habit memory (for review, see Goodman et al., 2012). Some researchers have proposed that these PTSD symptoms may be partially attributed to the effects of emotional arousal, i.e., stress stemming from the traumatic event, on the hippocampus and dorsolateral striatum (Packard, 2009; Schwabe et al., 2010b; Goodman et al., 2012).

Although the emotional modulation of memory observed in laboratory settings resembles the development of some PTSD symptoms, efforts to associate experimental findings with PTSD are limited by the stimuli and procedures used for eliciting emotional arousal. Experiments specifically examining the influence of stress/anxiety on hippocampus- and DLS-dependent memory have employed a variety of acute and chronic stressors, including predator odor (Leong and Packard, 2014), restraint stress (e.g., Kim et al., 2001; Sadowski et al., 2009; Taylor et al., 2014), fear-conditioned stimuli (e.g., Leong et al., 2015; Goode et al., 2016), and anxiogenic drugs (e.g., Packard and Wingard, 2004; Wingard and Packard, 2008; Schwabe et al., 2010a; Leong et al., 2012; Goodman et al., 2015). Although these stimuli engender robust increases in emotional arousal, the effects are presumably less severe than a traumatic event that produces PTSD symptoms. This limitation curbs the extent to which such laboratory models may be employed for understanding the development of PTSD and for designing novel treatments for the disorder.

In contrast to the stressors employed in previous studies examining the emotional modulation of memory systems, the single-prolonged stress (SPS) protocol produces a neurobehavioral profile that shares many features with PTSD (for review, see Yamamoto et al., 2009). Rats exposed to SPS display multiple behaviors that resemble PTSD symptoms, including sleep disturbances, exaggerated startle response, and impaired extinction of conditioned fear, among others (Liberzon et al., 1997; Khan and Liberzon, 2004; Imanaka et al., 2006; Yamamoto et al., 2009; Nedelcovych et al., 2015). In the present study, we examined whether SPS influences hippocampus-dependent spatial memory or DLS-dependent habit memory in a manner similar to acute and chronic stressors. Rats were initially subjected to SPS and then 1 week later received training in one of two distinct plus-maze tasks, i.e., a place learning task that invokes hippocampus-dependent spatial memory or a response learning task involving DLS-dependent habit memory. Following initial acquisition, rats also received extinction training to determine whether the impairing effect of SPS on fear extinction reported in previous studies (Liberzon et al., 1997; Yamamoto et al., 2008; Knox et al., 2012) may also be observed for spatial and habit memory.

## Materials and Methods

### Subjects

The present study employed 24 experimentally naïve male Sprague-Dawley rats weighing 350–450 g (Taconic, Hudson, NY, United States). Subjects were housed individually in a temperature-controlled vivarium with a 12 h light-dark cycle (lights on at 7:00 AM). Rats were food-restricted and reduced to 85% of their ad lib weight before the start of maze training. Rats were maintained at this weight throughout training, and water remained freely available in their home cages throughout the study. Animal use in this study was carried out in accordance with the ethical guidelines of the Institutional Animal Care and Use Committee (IACUC) at the University of Texas at Dallas. The protocol was approved by IACUC.

### Single-Prolonged Stress (SPS) Procedure

The procedure for SPS was selected based on previous studies examining the effects of SPS on PTSD-like symptoms in rats (e.g., Liberzon et al., 1997; Kohda et al., 2007; Yamamoto et al., 2008; Vanderheyden et al., 2015; for review see, Yamamoto et al., 2009). Rats were initially restrained for 2 h in a plastic cone. Immediately thereafter, they were placed into a circular pool of water (22 inch diameter, 25°C) for 20 min of forced swim. Rats were subsequently allotted a 15-min recuperation period before being exposed individually to diethyl ether vapor (Sigma) until they were anesthetized and unresponsive. Finally, rats were returned to their home cages and remained there for 7 days before the start of maze training.

### Maze Apparatus

The present study employed a plus-maze apparatus to examine hippocampus-dependent spatial memory and DLS-dependent habit memory. The apparatus consisted of four arms (23 cm in length, 5.75 cm in width, and 9.6 cm in height) arranged in a cross (+) orientation. A movable piece of Plexiglas was also used to block entry to the arm opposite to the start arm for each trial, creating a T-maze configuration that could be modified between trials. The maze was positioned in the center of a distinct room (separate from the room used for SPS) and was surrounded by various extra-maze visual cues.

### Training Procedures

The present training procedures were selected based on extensive previous work examining the influence of stress and anxiety on place and response learning in the plus-maze (e.g., Packard and Wingard, 2004; Wingard and Packard, 2008; Sadowski et al., 2009; Leong et al., 2012, 2015; Goodman et al., 2015). Seven days following the SPS procedure, rats were first habituated to the plus-maze apparatus for 2 days. For each day of habituation, rats were placed into the start arm (i.e., the North arm for the 1st day and the South arm for the 2nd day) and were allotted 5 min to explore the maze. Immediately following each day of habituation, rats were returned to their home cages with 3 Froot Loops cereal pieces (Kellogs). Rats were monitored to confirm Froot Loop consumption.

Twenty-four hours following habituation, separate groups of rats received maze training in either a place learning task (Experiment 1) or a response learning task (Experiment 2) for 12 days (6 trials/day). For each trial, rats were placed into the start arm (North or South) and had the opportunity to retrieve 1/2 Froot Loop in an opaque food cup at the end of the goal arm (East or West). The start arm sequence was NSSNNS on odd days (Days 1, 3, 5, etc.) and SNNSSN on even days (Days 2, 4, 6, etc.). After reaching the correct food cup and consuming the Froot Loop, or after 120 s had elapsed, the rat was removed from the maze. For the intertrial interval (ITI; 30 s), the rat was placed in a holding cage which was located behind a curtain, preventing the rat from viewing the maze during the ITI. For each trial, a correct response was recorded if the rat made an initial full-body entry into the arm containing the food, and an incorrect response was recorded if the animal entered the arm that did not contain the food. The proportion of correct turning responses over the course of training served as a measure of acquisition.

In experiment 1, rats previously given SPS (*n* = 6) or no stress (*n* = 6) were trained in a place learning task involving hippocampus-dependent spatial memory (Packard, 1999; Schroeder et al., 2002; Chang and Gold, 2003; Colombo et al., 2003; Compton, 2004; Jacobson et al., 2012). For each trial of the place learning task, food reinforcement was located in a consistent spatial location (i.e., in the food cup at the end of the West arm). Thus, rats acquired a spatial cognitive map of the learning environment to guide behavior from various start arms to the correct spatial location.

In experiment 2, a separate group of rats was trained in a response learning task invoking DLS-dependent habit memory (Packard and McGaugh, 1996; Chang and Gold, 2004; Palencia and Ragozzino, 2005; Asem and Holland, 2015; for reviews see, Packard and Goodman, 2017). Half the rats were previously given SPS (*n* = 6), and the other half received no stress procedures (i.e., the control group; *n* = 6). For each trial of the response learning task, food was located in the either the East or West goal arm depending on the rat’s starting position. When rats started from the North arm, food reinforcement could be found in the East arm, and when rats started from the South arm, food reinforcement was in the West arm. Thus, regardless of their starting position, rats were reinforced to acquire a consistent left turn response at the maze intersection to quickly obtain reinforcement. Previous evidence indicates that stress/anxiety impairs memory in the place and response learning tasks (Packard and Wingard, 2004; Wingard and Packard, 2008; for recent review, see Goodman et al., 2017a).

Twenty-four hours following the last day of acquisition, rats received extinction training (5 days for Experiment 1, and 4 days for Experiment 2). Extinction training was conducted in a manner identical to initial acquisition in each task, except the maze no longer contained food reinforcement. The proportion of correct turning responses over the course of extinction training served as a measure of extinction.

## Results

### Experiment 1: Place Learning Task

**FIGURE 1:**
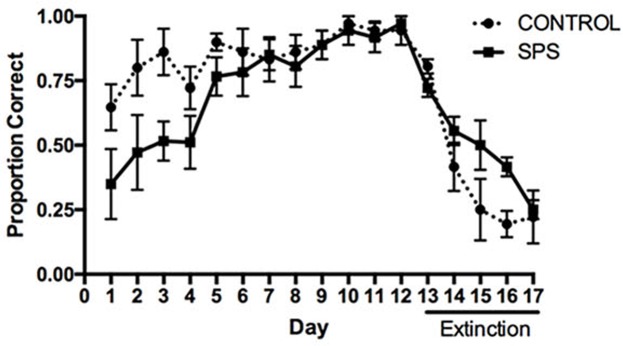
Influence of single-prolonged stress (SPS) on acquisition and extinction of hippocampus-dependent place learning in the plus-maze. SPS rats displayed a lower proportion of correct turning responses during acquisition (Days 1–12), relative to control rats, indicating an impairment of place learning. SPS rats were also slower to reduce the proportion of correct turning responses during extinction training (Days 13–17), indicating an impairment in extinction of place learning.

All statistical procedures were conducted using GraphPad Prism 7. The influence of SPS on acquisition and extinction in the place learning task is depicted in **Figure 1**. A repeated measures ANOVA analyzing the proportion of correct responses between the SPS and control groups over the course of acquisition indicated a main effect of Group [*F*(1,10) = 6.353, *p* = 0.030], main effect of Day [*F*(11,110) = 7.667, *p* < 0.000], and a trend toward a significant Group × Day interaction [*F*(11,110) = 1.670, *p* = 0.089]. Tests of simple main effects using Fisher’s LSD indicated that the proportion of correct turning responses differed significantly between the SPS and control groups on Training Days 1–3 (*p* < 0.05), whereas the groups did not differ significantly on Days 4–12 (*p* > 0.05). These findings suggest that early in training rats previously receiving SPS were impaired in acquisition of place learning, relative to rats in the control group; however, later in training, SPS and control rats showed comparable memory performance.

A repeated measures ANOVA analyzing the proportion of correct turning responses during extinction indicated a main effect of Day [*F*(4,40) = 20.99, *p* < 0.0001], but no main effect of Group [*F*(1,10) = 2.959, *p* = 0.1162]. However, there was a trend toward a significant Group × Day interaction [*F*(4,40) = 2.372, *p* = 0.0685]. Tests of simple main effects using Fisher’s LSD indicated that the proportion of correct turning responses differed between the SPS and control groups during extinction training on Days 15 and 16 (*p* < 0.05). These findings suggest that animals previously receiving SPS were slightly impaired in extinction of place learning, relative to the control group.

### Experiment 2: Response Learning Task

**FIGURE 2:**
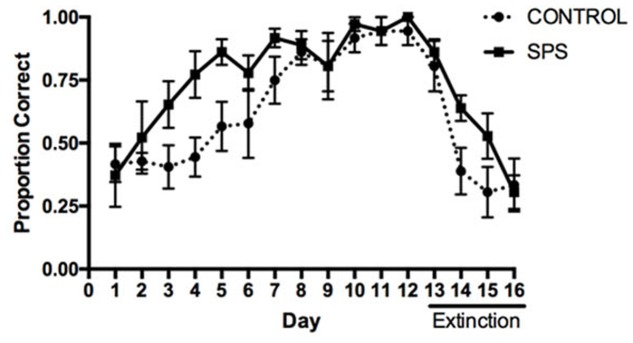
Influence of SPS on acquisition and extinction of dorsolateral striatum (DLS)-dependent response learning in the plus-maze. SPS rats demonstrated a greater proportion of correct turning responses during acquisition of response learning, compared to control rats, indicating an enhancement of response learning. In contrast, SPS rats were slower to reduce the number of correct turning responses during extinction training (Days 13–16), indicating that extinction of response learning was impaired.

The influence of SPS on acquisition and extinction in the response learning task is depicted in **Figure 2**. A repeated measures ANOVA analyzing the proportion of correct responses between the SPS and control groups over the course of acquisition indicated a main effect of Group [*F*(1,10) = 6.627, *p* = 0.0277], main effect of Day [*F*(11,110) = 12.11, *p* < 0.0001], and no Group × Day interaction [*F*(11,110) = 1.226, *p* = 0.2783]. Tests of simple main effects using Fisher’s LSD indicated that the proportion of correct turning responses differed significantly between the SPS and control groups on Training Days 3–5 (*p* < 0.05), whereas groups did not differ significantly from each other on Days 1–2 or 6–12 (*p* > 0.05). These findings indicate that rats previously receiving SPS demonstrated an enhancement in the acquisition of response learning, relative to control rats. This enhancement was mainly observed early in acquisition, whereas the SPS and control rats demonstrated comparable performance later in training.

A repeated measures ANOVA analyzing the proportion of correct turning responses during extinction indicated a main effect of Day [*F*(3,30) = 33.32, *p* < 0.0001], but no main effect of Group [*F*(1,10) = 1.570, *p* = 0.2387]. However, we did observe a significant Group × Day interaction [*F*(3,30) = 2.974, *p* = 0.0473]. Tests of simple main effects using Fisher’s LSD indicated that the proportion of correct turning responses only differed between the SPS and control groups during extinction training on Day 14 (*p* = 0.0439), whereas there was a trend for a difference on Day 15 (*p* = 0.0717). These results suggest that, relative to the control group, rats previously receiving SPS were impaired in extinction of response learning.

## Discussion

The present experiments suggest that multiple memory systems demonstrate different levels of functioning in a rat model of PTSD. Specifically, the SPS protocol, which produces PTSD-like symptoms in rats, may either enhance or impair learning depending on the type of memory being acquired. In Experiment 1, rats exposed to SPS demonstrated impaired acquisition of place learning in the plus-maze, relative to control rats. In contrast, in Experiment 2, rats given SPS showed enhanced acquisition of response learning, compared to controls. Despite differences in initial acquisition of place and response learning, SPS rats showed slower extinction relative to control rats in both the place and response learning tasks, suggesting a general impairment in extinction learning.

Extensive previous evidence indicates that acquisition of place and response learning depend on distinct memory systems. Place learning is mediated by hippocampus-dependent spatial memory, whereas response learning is mediated by DLS-dependent habit memory (for review, see White et al., 2013). Thus, the influence of SPS on performance in these tasks may be attributed to its modulation of hippocampus- and/or DLS-dependent memory systems. Importantly, the place and response learning tasks share similar non-mnemonic (e.g., motivational, motor, sensory, etc.) processes. Therefore, the differential influence of SPS on acquisition in the place and response learning tasks cannot be explained by a potential effect of SPS on non-mnemonic factors.

The present findings are in agreement with previous literature regarding the effects of emotional arousal on multiple memory systems. Acute and chronic behavioral stressors, as well as anxiogenic drugs, typically enhance hippocampus-dependent spatial/cognitive memory, while facilitating DLS-dependent habit memory (for reviews, see Packard and Goodman, 2012, 2013; Schwabe, 2013; Goldfarb and Phelps, 2017; Goodman et al., 2017a). The present results provide evidence that a prolonged stress protocol that produces PTSD-like symptoms in rats may influence memory systems in a similar manner. The present study is also consistent with prior evidence that SPS produces spatial memory impairments in the Morris water maze (e.g., Harvey et al., 2003; Kohda et al., 2007; Wang et al., 2010). It should be noted that other animal models of PTSD have been associated with long-term effects on memory systems (Baratta et al., 2007, 2008; Andero et al., 2011, 2012). Whether these alternative models influence spatial and habit memory in a manner similar to SPS should be examined in future research.

Although we did not examine the mechanisms by which SPS modulates these memory systems, the basolateral complex of the amygdala (BLA) may be involved. The BLA has been implicated in emotional modulation of hippocampus and DLS-dependent memory (Packard and Wingard, 2004; Wingard and Packard, 2008), and evidence indicates that SPS is associated with heightened BLA neural activity (Knox et al., 2016), as well as increased dendritic arborizations of BLA pyramidal neurons (Cui et al., 2008). Increased BLA activity may impair spatial memory via disrupting hippocampal synaptic plasticity (Akirav and Richter-Levin, 1999), i.e., a putative neural substrate of spatial learning (Martin et al., 2000). SPS also leads to downregulation of NMDA receptors in the hippocampus (Harvey et al., 2004), and this mechanism might explain the impairment in spatial memory in the present study, considering that NMDA receptor activation is required for acquisition of place learning in the plus-maze (Mackes and Willner, 2006; Watson and Stanton, 2009; Pol-Bodetto et al., 2011).

It is also possible that SPS influences the function of multiple memory systems via modulating the competitive interaction between hippocampus- and DLS-dependent memory (Poldrack and Packard, 2003). Competitive interactions between memory systems may be observed when disrupting the function of one system facilitates memory acquisition mediated by another system. For instance, several studies indicate that in some learning situations temporary or permanent lesion of the hippocampus leads to faster acquisition of habit memory (Packard et al., 1989; Kim et al., 2001; Schroeder et al., 2002). Moreover, several studies suggest that stress and anxiety may similarly facilitate habit memory via impairing hippocampal memory function (Wingard and Packard, 2008; for reviews, see Packard, 2009; Packard and Goodman, 2012, 2013; Goldfarb and Phelps, 2017). Considering that in the present study SPS impaired acquisition of place learning, and that this SPS protocol has also been associated with spatial memory impairments in other studies (Harvey et al., 2003; Kohda et al., 2007; Wang et al., 2010), disruption of hippocampal memory may constitute part of the mechanism allowing SPS to enhance DLS-dependent response learning in the plus-maze. Future research should investigate the precise neural mechanisms through which SPS modulates memory in the place and response learning tasks, in particular the role of the BLA, hippocampus, and DLS.

The differential influence of emotional arousal on memory systems is purportedly relevant to understanding the etiology of some clinical disorders, in particular those involving stress/anxiety, habit-like behavioral symptoms, and cognitive memory deficits. Disorders discussed in this context include obsessive-compulsive disorder, drug addiction, autism spectrum disorders, Tourette’s syndrome, and PTSD, among others (White, 1996; Packard, 2009; Schwabe et al., 2011; Goh and Peterson, 2012; Goodman et al., 2012, 2014; Everitt and Robbins, 2013; Gillan and Robbins, 2014). However, previous studies have employed stressors that neglect important aspects of PTSD pathology. The present study demonstrates that impaired spatial memory and enhanced habit memory may also be observed in a validated rat model of PTSD.

Importantly, the present findings are consistent with some features of PTSD. Indeed, PTSD has been associated with reduced hippocampal volume (Bonne et al., 2001; De Bellis et al., 2001; Fennema-Notestine et al., 2002; Pederson et al., 2004; Golier et al., 2005; Bremner, 2007), as well as impairments in hippocampus-dependent declarative memory (for review, see Bremner, 2006). Likewise, subjects with PTSD show differences in the structure and activity of the dorsal striatum, relative to healthy controls (for review, see Goodman et al., 2012), as well as greater connectivity between the hippocampus and striatum (Rangaprakash et al., 2017). Investigators have suggested that heightened avoidance to trauma-related cues may be regarded as an augmented, maladaptive form of S-R habit memory (Packard, 2009; Goodman et al., 2012). Moreover, subjects with PTSD demonstrate a greater likelihood of developing habit-like comorbidities, such as drug addiction and alcoholism (Jacobsen et al., 2001; McCauley et al., 2012). Whether avoidance symptoms and comorbid drug addiction/alcoholism are related to an enhancement of DLS-dependent habit memory in PTSD remains unknown.

Post-traumatic stress disorder subjects also show impairments in extinction of conditioned fear (Wessa and Flor, 2007; Milad et al., 2008), and this finding has been replicated in rats following exposure to SPS (Yamamoto et al., 2008; Knox et al., 2016). The present findings suggest that extinction of hippocampal spatial memories and DLS habit memories may also be impaired in this rat model of PTSD, even though initial learning of the spatial task was impaired and initial learning of the habit task was enhanced. Downregulation of NMDA receptors in the hippocampus following SPS (Harvey et al., 2004) may explain the impairment in spatial memory extinction, given that this kind of learning is dependent on activation of hippocampal NMDA receptors (Goodman et al., 2016). Extinction of response learning is mediated by the DLS and similarly involves NMDA receptor activity (Goodman et al., 2016, 2017b), however, the influence of SPS on NMDA receptor function and expression in the DLS has not been examined. In addition, whether extinction of spatial and habit memory is impaired in PTSD subjects will also require further investigation.

The persistence of stress and avoidance in PTSD may be due to the enhancement of initial learning or the failure to extinguish learned associations and responses. The present findings indicate that DLS-dependent habit learning is enhanced in a rat model of PTSD, suggesting that conditioned responses may be more difficult to overcome after experiencing a trauma. Results also suggest that extinction of appetitive learning is impaired, independent of whether the initial learning was enhanced or not. These findings are consistent with others demonstrating impairments in the extinction of contextual and auditory fear conditioning (Yamamoto et al., 2008; Knox et al., 2012), and they show that extinction impairments are not specific to fear learning in the rat model of PTSD.

## Ethics Statement

This study was carried out in accordance with the recommendations of the National Institutes of Health’s Office of Laboratory Animal Welfare. The protocol was approved by the Institutional Animal Care and Use Committee at the University of Texas at Dallas.

## Author Contributions

The research was carried out by JG when he was a *post doc* in CM’s lab. The study was designed by both authors. JG did the data analysis and wrote the first draft of the manuscript. CM edited and added to the manuscript.

## Conflict of Interest Statement

The authors declare that the research was conducted in the absence of any commercial or financial relationships that could be construed as a potential conflict of interest.
